# Nanoscale Structural Characterization of Amyloid β 1–42 Oligomers and Fibrils Grown in the Presence of Fatty Acids

**DOI:** 10.1021/acschemneuro.4c00275

**Published:** 2024-09-02

**Authors:** Kiryl Zhaliazka, Dmitry Kurouski

**Affiliations:** † Department of Biochemistry and Biophysics, Texas A&M University, College Station, Texas 77843, United States; ‡ Department of Biomedical Engineering, Texas A&M University, College Station, Texas 77843, United States

**Keywords:** amyloid β_1−42_, polyunsaturated fatty acids, oligomers, fibrils, AFM-IR

## Abstract

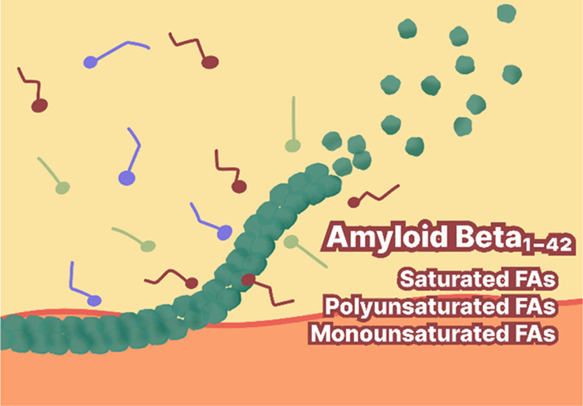

Mono- and polyunsaturated fatty acids (FAs) are broadly used as food supplements. However, their effect on the aggregation of amyloidogenic proteins remains unclear. In this study, we investigated the effect of a large number of mono- and polyunsaturated, as well as fully saturated FAs on the aggregation of amyloid β_1–42_ (A_β1–42_) peptide. A progressive aggregation of this peptide is the expected molecular cause of Alzheimer’s disease (AD), one of the most common neurodegenerative pathologies in the world. We found that arachidonic and stearic acids delayed the aggregation of Aβ1–42. Using Nano-Infrared spectroscopy, we found that FAs caused very little if any changes in the secondary structure of A_β1–42_ oligomers and fibrils formed at different stages of protein aggregation. However, the analyzed mono- and polyunsaturated, as well as fully saturated FAs uniquely altered the toxicity of Aβ1–42 fibrils. We found a direct relationship between the degree of FAs unsaturation and toxicity of Aβ1–42 fibrils formed in their presence. Specifically, with an increase in the degree of unsaturation, the toxicity A_β1–42_/FA fibrils increased. These results indicate that fully saturated or monounsaturated FAs could be used to decrease the toxicity of amyloid aggregates and, consequently, decelerate the development of AD.

## Introduction

Amyloid β_1–42_ (Aβ_1–42_) peptide is the major component of amyloid plaques, extracellular formations that are commonly observed in the gray matter of patients diagnosed with Alzheimer’s disease (AD).^1,2^ This peptide is produced by γ-secretases from amyloid precursor protein.^3−6^ Numerous *in vitro* studies demonstrated that free Aβ_1–42_ rapidly aggregates at physiological conditions producing oligomers and fibrils.^7−11^ These highly toxic aggregates can spread across the brain causing progressive neurodegeneration^12−15^

The toxicity of Aβ aggregates, as well as the rate of peptide aggregation, can be altered by a large number of biological molecules.^16,17^^18−20^ For instance, Chan and co-workers showed that octahedral cobalt complexes with polyaromatic ligands were able to inhibit the aggregation of Aβ peptide due to dual binding mode involving π–π stacking and metal coordination to amino acids of the peptide.^21^ Similar effects were reported for The paddlewheel [Ru_2_Cl(O_2_CCH_3_)_4_] complex by Terran and co-workers on lysozyme,^22^ while La Manna and co-workers demonstrated that metal complexes could alter aggregation of peptides via release of carbon monoxide.^23^ Aromadendrin, on the opposite accelerated amyloid aggregation and fibril formation simultaneously reducing neuroblastoma and insulinoma toxicity of Aβ_42_ aggregates.^24^ Our group demonstrated that cholesterol and saturated phospholipids strongly enhanced the rate of Aβ_1–42_ aggregation.^25^ Furthermore, the presence of 5% cholesterol relative to phosphatidylcholine (PC) in large unilamellar vesicles (LUVs), did not change the rate of oligomer formation. However, the propagation of such oligomers into fibrils was observed, which was not evident for PC alone. It was also demonstrated that Aβ_1–42_ fibrils formed in the presence of cardiolipin (CL), PC, and PC/cholesterol mixture exerted much greater levels of cell toxicity compared to the aggregates formed in the lipid-free environment.^25^

Utilization of Nano-Infrared spectroscopy revealed that cytotoxicity of Aβ_1–42_ fibrils had a direct relationship with the amount of parallel β-sheet in these aggregates.^25^ In Nano-Infrared, also known as atomic force microscopy Infrared (AFM-IR) spectroscopy, a metalized scanning probe can be placed directly at the oligomer or fibril deposited onto a silicon wafer.^26−30^ Next, pulsed tunable IR light is used to cause thermal expansions in the aggregates that are recorded by the scanning probe and converted into IR spectra.^31−34^ In the acquired spectra, the amide I band (1600–1700 cm^–1^) can be used to reveal the secondary structure of protein aggregates.^35^ The presence of amide I around 1630 cm^–1^ indicates the predominance of parallel β-sheet, whereas its shift to 1660 cm^–1^ indicates the presence of an unordered protein secondary structure.^33,36^ Finally, the antiparallel β-sheet exhibits a much higher frequency in the IR spectra (1695 cm^–1^), which allows for its differentiation from the parallel β-sheet.^33,36,37^

Recently reported results by our group showed that LUVs with unsaturated phospholipids caused changes in the secondary structure of Aβ_1–42_ oligomers and fibrils.^38^ Furthermore, if present at the stage of peptide aggregation, LUVs with unsaturated PC, CL, and phosphatidylserine (PS) reduce the toxicity of Aβ_1–42_ fibrils. Similar results were recently reported by Eto and co-workers for Aβ_1–40_ fibrils formed in the presence of docosahexaenoic acid (DHA).^39^ At the same time, this polyunsaturated fatty acid (FA), drastically increased the toxicity of insulin fibrils.^40^ Thus, one can expect that the effect of FAs on the toxicity of protein aggregates directly depends on the structure of the amyloid protein.

Eto and co-workers also showed that DHA could increase the aggregation rate of Aβ_1–40_, which was not observed for palmitic acid.^39^ These results suggest that FAs can not only change the toxicity of Aβ aggregates but also alter the aggregation rate of Aβ peptides. Expanding upon this, we investigated the effect of several poly and monounsaturated FAs on the rate of Aβ_1–42_ aggregation. We also used AFM-IR to examine the secondary structure of Aβ_1–42_ oligomers and fibrils formed at the early and late stages of protein aggregation. Finally, we tested the cytotoxicity of amyloid aggregates using the N27 rat dopaminergic cell line.

## Results

### Elucidation of the Role of FAs on the Aggregation Rate of Aβ_1–42_

**Figure 1:**
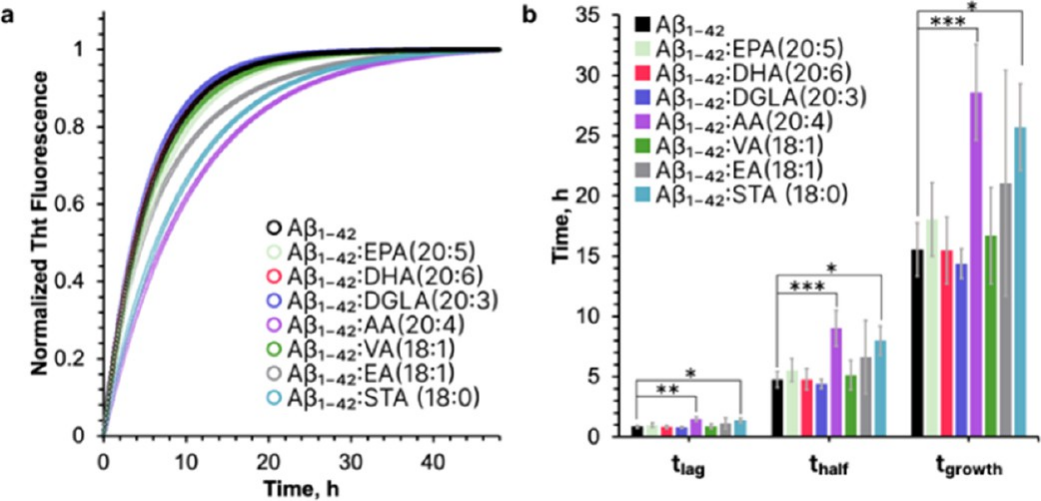
Influence of FAs on the ThT Aggregation Kinetics of Aβ_1–42_. (a) The aggregation kinetics of Aβ_1–42_ in the presence of different FAs, as indicated by normalized Thioflavin T fluorescence. Kinetic curves were fitted using equations from Table S1. (b) The bar graphs show quantified time parameters for Aβ_1–42_ aggregation kinetics: lag time (*t*_lag_), half-time (*t*_half_), and growth phase (*t*_growth_), with each condition tested against the control (Aβ_1–42_ along) using one-way ANOVA and Turkey’s HSD test, with statistical significance indicated (*p < 0.05, **p < 0.01, ***p < 0.001).

We first investigated the extent to which FAs, including polyunsaturated, monounsaturated, and saturated, alter the aggregation rate of Aβ_1–42_, Figure 1a. Specifically, we tested the effect of polyunsaturated FAs like Eicosapentaenoic Acid (EPA (20:5)) and Docosahexaenoic Acid (DHA (20:6)), Dihomo-γ-Linolenic Acid (DGLA (20:3)), and Arachidonic Acid (AA (20:4)), as well as monounsaturated FAs such as Elaidic Acid (EA (18:1)) and Vaccenic Acid (VA (18:1)). We also compared these results to the effect exerted by a fully saturated Stearic Acid (STA (18:0)). For this, the aggregation kinetics of Aβ_1–42_ in the presence and absence of the dissed above FAs were analyzed using Thioflavin T fluorescence assay. Next, we determined *t*_lag_, *t*_half_, and *t*_growth_ values that represented 10% (*t*_lag_), 50% (*t*_half_), and 90% (*t*_growth_) of the maximum ThT fluorescence intensity, Figure 1b.

In the case of Aβ_1–42_ alone, the *t*_lag_ was found to be 0.87 ± 0.11 h, *t*_half_ was 4.77 ± 0.65 h, and *t*_growth_ was 15.55 ± 2.20 h, Figure 1 and Table 1. The introduction of FAs showed varied impacts on these parameters. For Aβ_1–42_/DGLA (20:3), the *t*_lag_ was 0.80 ± 0.06 h, *t*_half_ was 4.42 ± 0.38 h, and *t*_growth_ was 14.08 ± 1.21 h. This indicates a slight but not significant decrease in the aggregation rate of Aβ_1–42_ compared to the control. For Aβ_1–42_/AA (20:4), significant changes were observed: *t*_lag_ increased to 1.50 ± 0.21 h, *t*_half_ to 9.02 ± 1.47 h, and *t*_growth_ to 28.6 ± 3.96 h, indicating a substantial delay in Aβ_1–42_ aggregation. For Aβ_1–42_/DHA (20:6), we observed *t*_lag_ = 0.83 ± 0.12 h, *t*_half_ = 4.78 ± 0.87 h, and *t*_growth_ = 15.48 ± 2.77 h. These results showed that DHA has very little if any impact on the aggregation kinetics of Aβ_1–42_. For Aβ_1–42_/EA (18:1), the *t*_lag_ was 1.13 ± 0.46 h *t*_half_ was 6.62 ± 3.03 h, and *t*_growth_ was 21.07 ± 9.37 h, indicating a moderate delay in the protein aggregation. In samples with Aβ_1–42_/EPA (20:5), the *t*_lag_ was 0.98 ± 0.16 h, *t*_half_ was 5.55 ± 0.94 h, and *t*_growth_ was 18.05 ± 3.05 h, showing a slight delay in aggregation kinetics. Significant changes were observed in Aβ_1–42_/STA (18:0) samples, with a *t*_lag_ of 1.37 ± 0.20 h, *t*_half_ of 8.00 ± 1.21 h, and *t*_growth_ of 25.71 ± 3.6 h. Lastly, for Aβ_1–42_/VA (18:1), the *t*_lag_ was 0.88 ± 0.18 h, *t*_half_ was 5.15 ± 1.22 h, and *t*_growth_ was 16.7 ± 4 h, indicating a slight deceleration of Aβ_1–42_ aggregation. Based on these results, we can conclude that the presence of FAs at the stage of Aβ_1–42_ aggregation resulted in varying degrees of deceleration of the protein aggregation. Specifically, AA (20:4) and STA (18:0) caused a significant delay in the aggregation rate of Aβ_1–42_, whereas other FAs showed only small changes in the rate of protein aggregation. These variations in aggregation kinetics suggest a correlation with the structural properties of the aggregates formed, potentially implicating the role of specific FAs in modulating the aggregation pathway and structure of Aβ_1–42_.

**Table 1:** Kinetic Parameters of Aβ_1-42_ Aggregation Alone and in the Presence of FAs

sample	*t*_lag_, h	*t*_half_, h	*t*_growth_, h
Aβ_1–42_ alone	0.87 ± 0.11	4.77 ± 0.65	15.55 ± 2.20
Aβ_1–42_/DGLA	0.80 ± 0.06	4.42 ± 0.38	14.08 ± 1.21
Aβ_1–42_/AA	1.50 ± 0.21	9.02 ± 1.47	28.6 ± 3.96
Aβ_1–42_/DHA	0.83 ± 0.12	4.78 ± 0.87	15.48 ± 2.77
Aβ_1–42_/EA	1.13 ± 0.46	6.62 ± 3.03	21.07 ± 9.37
Aβ_1–42_/EPA	0.98 ± 0.16	5.55 ± 0.94	18.05 ± 3.05
Aβ_1–42_/STA	1.37 ± 0.20	8.00 ± 1.21	25.71 ± 3.6
Aβ_1–42_/VA	0.88 ± 0.18	5.15 ± 1.22	16.7 ± 4

### Nanoscale Imaging of Aβ_1–42_ Oligomers and Fibrils Formed in the Presence of FAs

**Figure 2:**
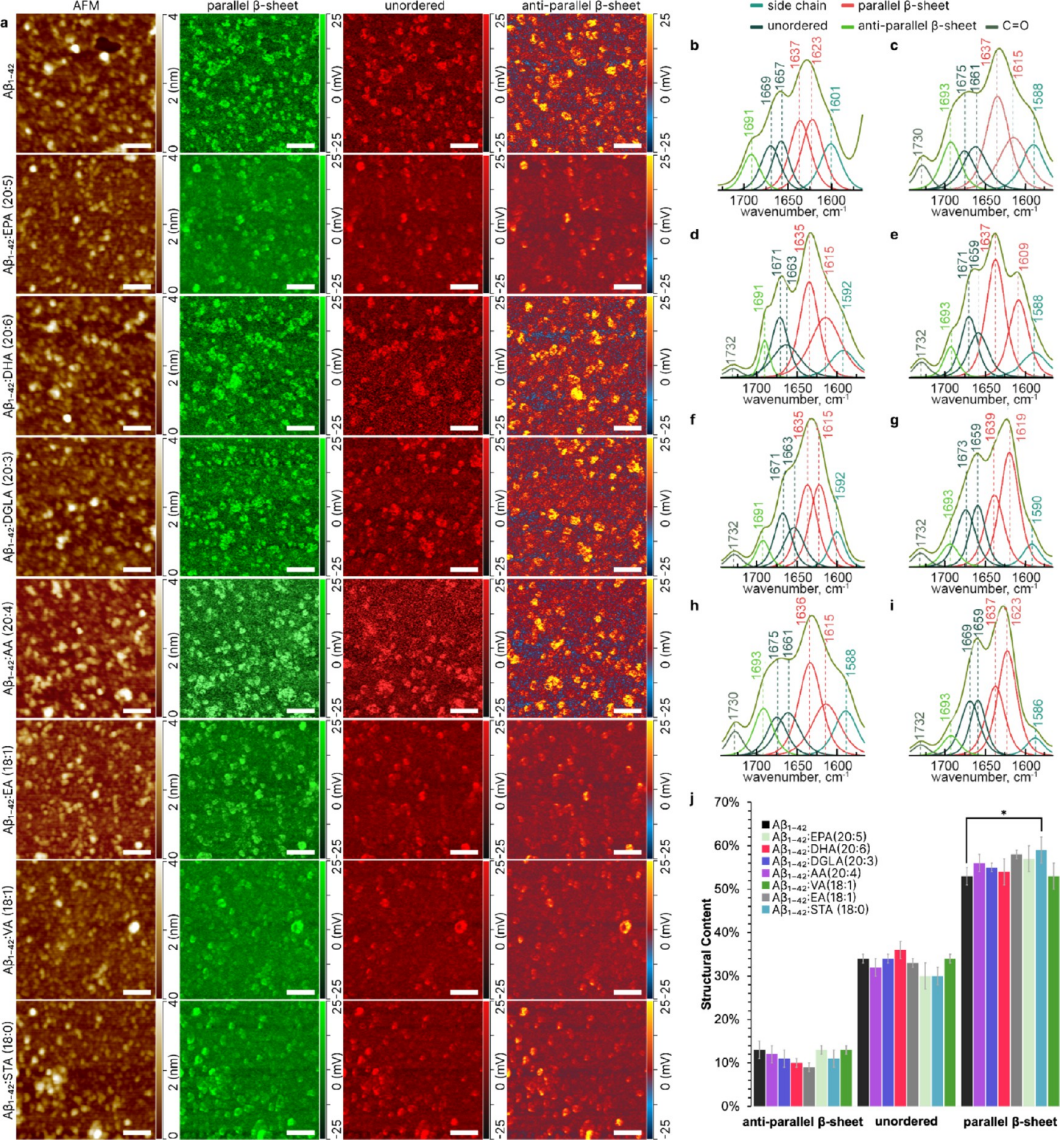
Structural Characterization of Aβ1–42 Oligomers Formed in the Presence and Absence of Different Fatty Acids at the 3 h of Protein Aggregation. (a) Combined atomic force microscopy (AFM) and Infrared (IR) mapping of Aβ1–42 aggregates formed in the presence or absence of various FAs, showcasing the presence of parallel (1630 cm^–1^), unordered (1650 cm^–1^), and antiparallel (1694 cm^–1^) β-sheet secondary structures. (b-i) IR spectral deconvolution for Aβ1–42 oligomers formed in the absence (b) or in the presence of EPA (20:5) (c), DHA (20:6) (d), DGLA (20:3) (e), AA (20:4) (f), VA (18:1) (g), EA (18:1) (h), and STA (18:0) (i). (j) Quantitative assessment of the secondary structure of Aβ1–42 oligomers. The spectra indicate the relative content of parallel β-sheet, unordered, and antiparallel β-sheet structures in protein oligomers. Data were analyzed using one-way ANOVA and Turkey’s HSD post hoc test for multicomparison (* *p* < 0.05).

**Figure 3:**
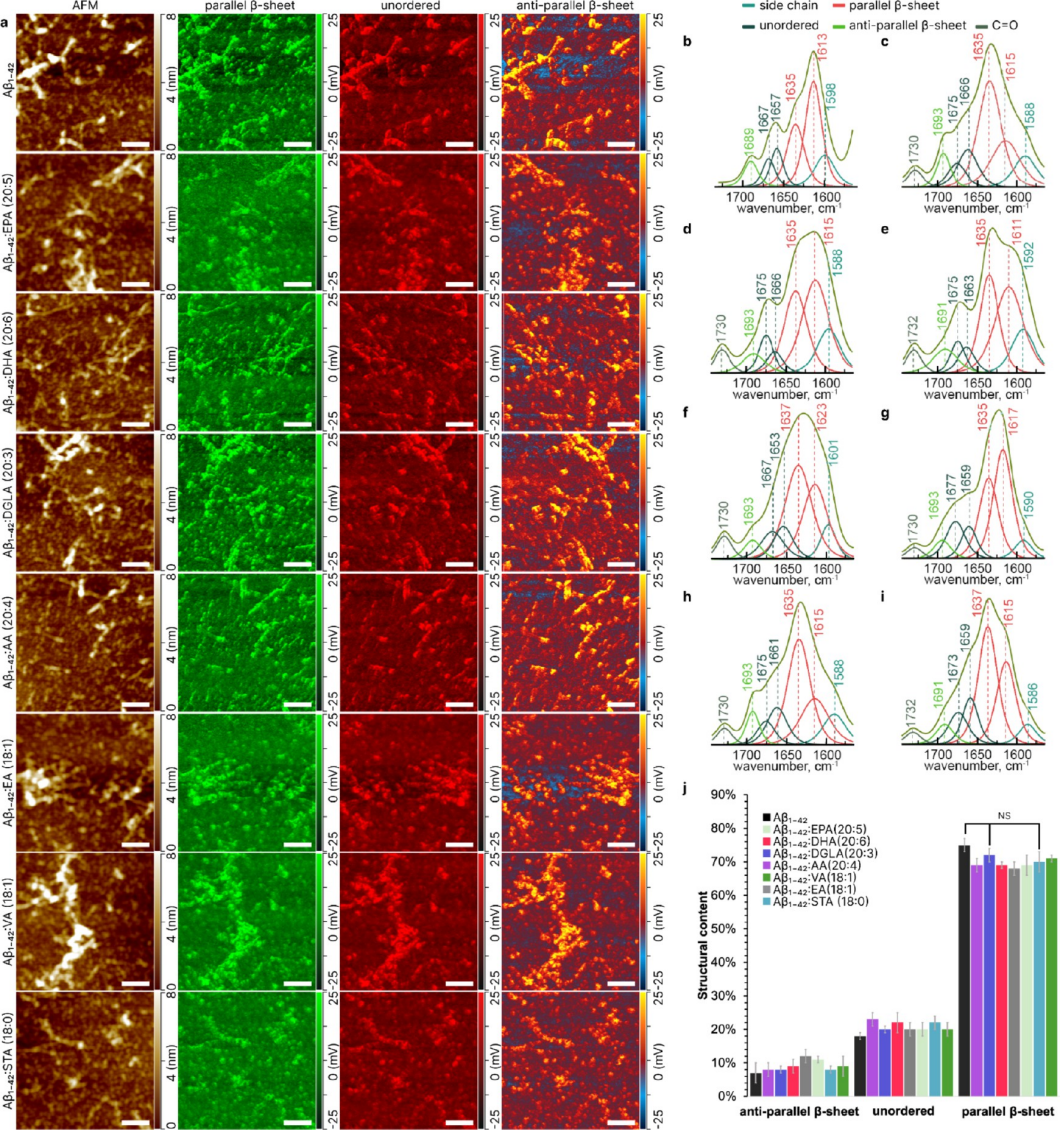
Structural Characterization of Aβ1–42 Fibrils Formed in the Presence and Absence of Different Fatty Acids at the 48 h of Protein Aggregation. (a) Combined atomic force microscopy (AFM) and Infrared (IR) mapping of Aβ1–42 aggregates formed in the presence or absence of various FAs, showcasing the presence of parallel (1630 cm^–1^), unordered (1650 cm^–1^), and antiparallel (1694 cm^–1^) β-sheet secondary structures. (b-i) IR spectral deconvolution for Aβ1–42 fibrils formed in the absence (b) or in the presence of EPA (20:5) (c), DHA (20:6) (d), DGLA (20:3) (e), AA (20:4) (f), VA (18:1) (g), EA (18:1) (h), and STA (18:0) (i). (j) Quantitative assessment of the secondary structure of Aβ1–42 oligomers. The spectra indicate the relative content of parallel β-sheet, unordered, and antiparallel β-sheet structures in protein fibrils. Data were analyzed using one-way ANOVA and Turkey’s HSD post hoc test for multicomparison (* *p* < 0.05).

We used atomic force microscopy (AFM) to investigate the morphology of protein aggregates formed at the early (3 h) (Figure 2a), and late (48 h), (Figure 3a) stages of protein aggregation. Morphological analysis of Aβ_1–42_ aggregates formed at the early stage revealed the presence of uniform round-shaped oligomers with heights of 2–3 nm. This observation was consistent in both Aβ_1–42_ alone and Aβ_1–42_ treated with a variety of FAs. In terms of secondary structure, as determined by Nano-Infrared (AFM-IR) spectroscopy, distinct patterns were observed. The Aβ_1–42_ exhibited a secondary structure composition of 13 ± 2% antiparallel β-sheet, 34 ± 1% unordered, and 53 ± 2% parallel β-sheet. Variations were noted in FA-treated samples: Aβ_1–42_/AA (20:4) showed 12 ± 2% antiparallel β-sheet, 32 ± 2% unordered, and 56 ± 2% parallel β-sheet; Aβ_1–42_/DGLA (20:3) had 11 ± 2% antiparallel β-sheet, 34 ± 1% unordered, and 55 ± 1% parallel β-sheet; Aβ_1–42_/DHA (20:6) presented 10 ± 1% antiparallel β-sheet, 36 ± 1% unordered, and 54 ± 2% parallel β-sheet;

Aβ_1–42_/EA (18:1) displayed 9 ± 1% antiparallel β-sheet, 33 ± 1% unordered, and 58 ± 2% parallel β-sheet; Aβ_1–42_/EPA (20:5) exhibited 13 ± 1% antiparallel β-sheet, 30 ± 3% unordered, and 57 ± 1% parallel β-sheet; Aβ_1–42_/STA (18:0) showed 11 ± 3% antiparallel β-sheet, 30 ± 1% unordered, and 59 ± 3% parallel β-sheet; and Aβ_1–42_/VA (18:1) had 13 ± 1% antiparallel β-sheet, 34 ± 1% unordered, and 53 ± 1% parallel β-sheet. These results indicate that the presence of round-shaped oligomers at the early stage is a consistent feature across all samples, with the secondary structure composition exhibiting slight but notable variations depending on the FA present at the stage of peptide aggregation.

**Figure 4:**
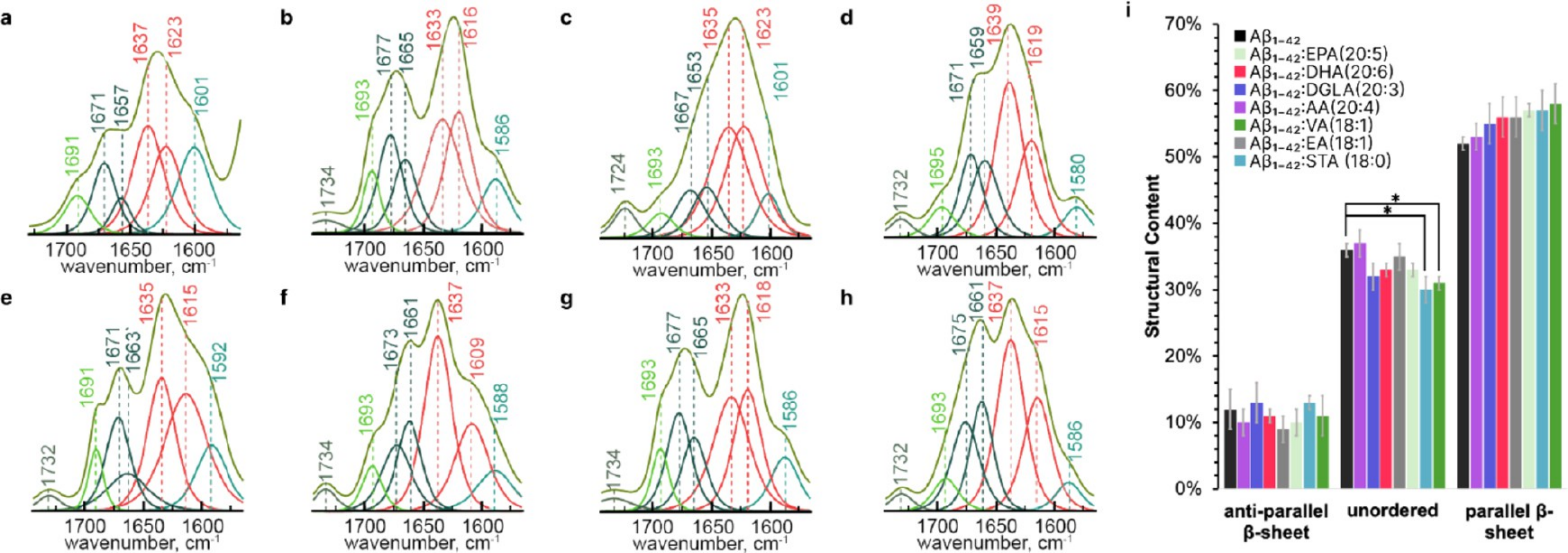
Structural Characterization of Aβ1–42 Fibrils Formed in the Presence and Absence of Different Fatty Acids at the 48 h of Protein Aggregation. (a) Combined atomic force microscopy (AFM) and Infrared (IR) mapping of Aβ1–42 aggregates formed in the presence or absence of various FAs, showcasing the presence of parallel (1630 cm^–1^), unordered (1650 cm^–1^), and antiparallel (1694 cm^–1^) β-sheet secondary structures. (b-i) IR spectral deconvolution for Aβ1–42 fibrils formed in the absence (b) or in the presence of EPA (20:5) (c), DHA (20:6) (d), DGLA (20:3) (e), AA (20:4) (f), VA (18:1) (g), EA (18:1) (h), and STA (18:0) (i). (j) Quantitative assessment of the secondary structure of Aβ1–42 oligomers. The spectra indicate the relative content of parallel β-sheet, unordered, and antiparallel β-sheet structures in protein fibrils. Data were analyzed using one-way ANOVA and Turkey’s HSD post hoc test for multicomparison (* *p* < 0.05).

At the late stage, 48 h we found both oligomers and fibrils in all samples, indicating a progression of Aβ_1–42_ oligomers into fibrils (Figure 3a). All observed oligomers, in both the control group (Aβ_1–42_ alone) and samples treated with various FAs, had a spherical appearance. AFM-IR revealed notable variations in the secondary structure among different samples (Figure 4). The Aβ_1–42_ oligomers exhibited a secondary structure composition of 12 ± 3% antiparallel β-sheet, 36 ± 1% unordered, and 52 ± 1% parallel β-sheet. In contrast, FA-treated samples showed slight but discernible variations in this pattern. Aβ_1–42_/AA (20:4) samples demonstrated 10 ± 2% antiparallel β-sheet, 37 ± 2% unordered, and 53 ± 1% parallel β-sheet. Aβ_1–42_/DGLA (20:3) contained 13 ± 2% antiparallel β-sheet, 32 ± 1% unordered, and 55 ± 2% parallel β-sheet. Aβ_1–42_/DHA (20:6) had 11 ± 1% antiparallel β-sheet, 33 ± 1% unordered, and 56 ± 2% parallel β-sheet. Aβ_1–42_/EA (18:1) showed 9 ± 1% antiparallel β-sheet, 35 ± 2% unordered, and 56 ± 3% parallel β-sheet. Aβ_1–42_/EPA (20:5) exhibited 10 ± 1% antiparallel β-sheet, 33 ± 2% unordered, and 57 ± 2% parallel β-sheet. Aβ_1–42_/STA (18:0) presented 13 ± 2% antiparallel β-sheet, 30 ± 1% unordered, and 57 ± 2% parallel β-sheet. Lastly, Aβ_1–42_/VA (18:1) had 11 ± 1% antiparallel β-sheet, 31 ± 2% unordered, and 58 ± 1% parallel β-sheet. These results indicate a trend of maintaining or slightly increasing the number of parallel β-sheets in FA-treated samples compared to the control, suggesting a potential influence of specific FAs on the structural evolution of Aβ_1–42_ oligomers. Intriguingly, our AFM-IR analysis revealed that the secondary structure of early and late-stage oligomers exhibited remarkable similarity. This observation implies that the secondary structure of the Aβ_1–42_ oligomers, characterized by the presence of parallel β-sheets, is established early in the aggregation process, and remains largely consistent over time.

At the late stage of protein aggregation, a critical phase in the aggregation process of Aβ_1–42_ was observed presence of elongated fibrils in all samples (Figure 3a), signifying an advanced stage of aggregation. This morphological change was consistent in both the control group (Aβ_1–42_ alone) and in samples treated with various FAs. In terms of secondary structure content, the control group exhibited a composition of 7 ± 3% antiparallel β-sheet, 18 ± 2% unordered, and 75 ± 2% parallel β-sheet. Notably, FA-treated samples demonstrated variations in this pattern: Aβ_1–42_/AA (20:4) had 8 ± 2% antiparallel β-sheet, 23 ± 2% unordered, and 69 ± 2% parallel β-sheet; Aβ_1–42_/DGLA (20:3) showed 8 ± 2% antiparallel β-sheet, 20 ± 2% unordered, and 72 ± 2% parallel β-sheet; Aβ_1–42_/DHA (20:6) contained 9 ± 3% antiparallel β-sheet, 22 ± 1% unordered, and 69 ± 3% parallel β-sheet; Aβ_1–42_/EA (18:1) had 12 ± 3% antiparallel β-sheet, 20 ± 2% unordered, and 68 ± 3% parallel β-sheet; Aβ_1–42_/EPA (20:5) exhibited 11 ± 2% antiparallel β-sheet, 20 ± 1% unordered, and 69 ± 2% parallel β-sheet; Aβ_1–42_/STA (18:0) presented 8 ± 2% antiparallel β-sheet, 22 ± 2% unordered, and 70 ± 3% parallel β-sheet; and Aβ_1–42_/VA (18:1) showed 9 ± 3% antiparallel β-sheet, 20 ± 2% unordered, and 71 ± 3% parallel β-sheet. These results highlight the predominance of parallel β-sheets in the fibrils across all samples. However, there were slight but noticeable variations in the secondary structure composition dependent on the specific FAs used. Interestingly, while the proportion of parallel β-sheets was slightly less in most FA-treated samples compared to the Aβ control, Dihomo-γ-Linolenic Acid (DGLA) and Vaccenic Acid (VA) samples showed a secondary structure composition similar to that of the control. This indicates a potential modulatory effect of FAs on the structural evolution of Aβ_1–42_ aggregates.

### Toxicity of Aβ_1–42_ Oligomers and Fibrils Formed in the Presence of FAs

**Figure 5:**
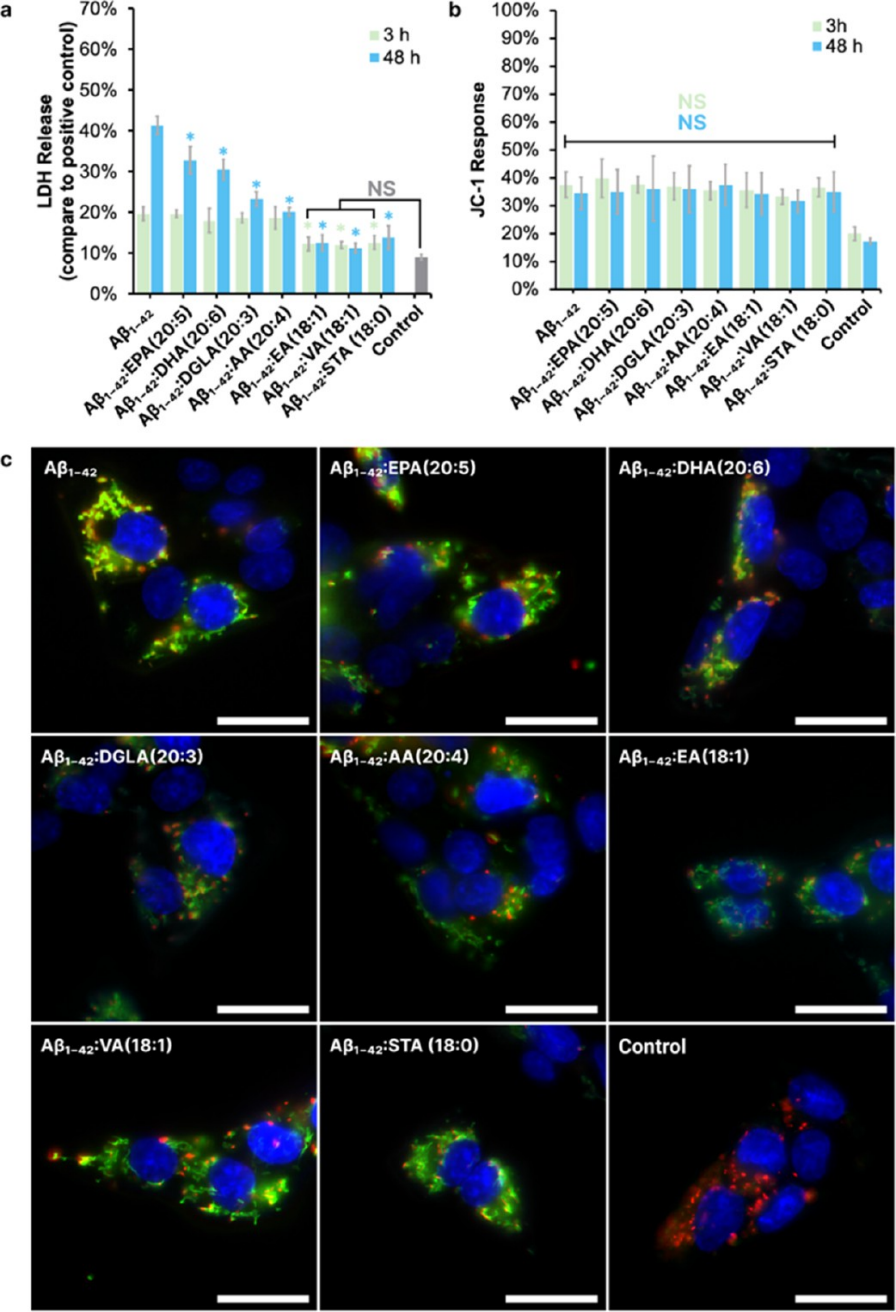
Effects of fatty acids on LDH release, mitochondrial membrane potential. (a) LDH release at 3- and 48 h post-treatment, indicating cytotoxicity levels. Statistical significance is indicated by asterisks (*p < 0.05), where green −3-h time point, blue–for 48-h time point related to Aβ_1–42_ along and nonsignificant differences are denoted as NS compared to control, determined by ANOVA followed by Tukey’s HSD test. (b) JC-1 assay results for mitochondrial membrane potential at the same time points, with nonsignificant changes marked as NS along samples incubated with FAs compared to Aβ_1–42_ along. (c) Fluorescence microscopy images showing neuronal cultures treated with Aβ_1–42_ peptides alone or in conjunction with specific FAs. Green fluorescence indicates areas of mitochondrial neurotoxicity, and blue shows neuronal nuclei. Scale bars 50 μm.

We used LDH assay to assess the impact of FAs on the toxicity that Aβ_1–42_ aggregates formed at the early and late stages of protein aggregation (Figure 5a). We found that early stage Aβ_1–42_ oligomers exerted an LDH level of 19.7 ± 1.7%, that is significantly higher than the control (9.0 ± 0.7%). Similarly, Aβ_1–42_ formed in the presence of EPA (20:5), DHA (20:6), DGLA (20:3), and AA (20:4) showed LDH levels of 19.7 ± 0.9, 18.0 ± 2.9, 18.7 ± 1.2, and 18.7 ± 2.6%, respectively, indicating no significant cytotoxic increase compared to Aβ_1–42_ oligomers formed in the FA-free environment. Notably, samples formed in the presence of EA (18:1), VA (18:1), and STA (18:0) demonstrated significant decreases in LDH levels to 12.3 ± 1.7, 12.0 ± 0.8, and 12.7 ± 1.7%, respectively. It should be noted that LDH assay revealed that FAs themselves exerted no noticeable cytotoxicity to N27 rat neurons (Figure S2a). This further supports the notion that the observed reduction in cytotoxicity is specifically related to the interaction between the FAs and the Aβ1–42 aggregates, rather than an intrinsic property of the FAs.

Contrastingly, late stage Aβ_1–42_ fibrils formed in the lipid-free environment showed LDH level of 41.3 ± 2.3%, while all FA-treated Aβ_1–42_ samples indicated significant reduction in cytotoxicity. The LDH levels in Aβ_1–42_/EPA, Aβ_1–42_/DHA (20:6), Aβ_1–42_/DGLA (20:3), Aβ_1–42_/AA (20:4), Aβ_1–42_/EA (18:1), Aβ_1–42_/VA, and Aβ_1–42_/STA were 32.8 ± 3.4, 30.5 ± 2.5, 23.3 ± 1.8, 20.2 ± 1.0, 12.6 ± 2.0, 11.3 ± 1.2, and 13.9 ± 2.8%, respectively. These results suggest a potential protective or modulatory influence of FAs on the cytotoxicity of Aβ_1–42_ aggregates, as evidenced by the consistent decrease in LDH release in FA-treated samples compared to Aβ_1–42_ fibrils formed in the FA-free environment.

To examine the effects of Aβ_1–42_ aggregates on mitochondrial (MT) activity in N27 rat dopaminergic cells exposed to these protein aggregates, we utilized a combination of the JC-1 assay, flow cytometry, and fluorescence microscopy. This investigation was conducted for Aβ_1–42_ aggregates formed at early and late stages of protein aggregation in the presence and absence of FAs.

We found that early stage Aβ_1–42_ oligomers exhibited JC-1 intensity of 37.45 ± 4.66%, which is significantly higher than the control (19.95 ± 2.47%). Aβ_1–42_ oligomers formed in the presence of FAs displayed similar intensities. Specifically, Aβ_1–42_/AA (39.64 ± 6.90%), Aβ_1–42_/STA (37.48 ± 3.07%), Aβ_1–42_/DHA (36.90 ± 4.94%), Aβ_1–42_/EA (35.37 ± 3.30%), Aβ_1–42_/DGLA (35.50 ± 6.29%), Aβ_1–42_/VA (33.22 ± 2.68%), and Aβ_1–42_/EPA (36.51 ± 3.34%) showed no significant differences in JC-1 intensity compared to the Aβ_1–42_ formed in the FA-free environment. Importantly, it should be noted that the FAs themselves, when tested independently, did not alter the JC-1 response, as indicated by separate tests (Figure S2b). These results indicate that all analyzed Aβ_1–42_ oligomer samples exert the same magnitude of mitochondrial impairment in N27 cells.

Aβ_1–42_ fibrils formed at the late stage showed JC-1 intensity of 34.30 ± 5.80%, which is significantly higher than the control (17.18 ± 1.20%). Similar intensities of JC-1 were observed for all other samples: Aβ_1–42_/AA (34.86 ± 7.96%), Aβ_1–42_/STA (36.06 ± 11.69%), Aβ_1–42_/DHA (35.90 ± 8.31%), Aβ_1–42_/EA (37.29 ± 7.35%), Aβ_1–42_/DGLA (34.11 ± 7.53%), Aβ_1–42_/VA (31.57 ± 4.05%), and Aβ_1–42_/EPA (34.87 ± 7.15%). These findings show that all analyzed Aβ_1–42_ fibril samples cause the same or similar magnitude of mitochondrial damage.

To corroborate the findings derived from the JC-1 assay conducted with flow cytometry, we conducted a fluorescence microscopy analysis on N27 cells exposed to Aβ_1–42_ fibrils grown in the presence and absence of FAs. The microscopy images unequivocally demonstrated a notable elevation in green JC-1 fluorescence intensity, signifying a decrease in mitochondrial membrane potential, in cells treated with all variants of Aβ_1–42_ fibrils in comparison to the control cells.

## Discussion

In our study, we investigated the effect of FAs with varying saturation levels on Aβ_1–42_ aggregation. Our findings reveal a complex interplay between the saturation level of FAs and the aggregation behavior of Aβ_1–42_, as well as its subsequent structural, cytotoxic, and mitochondrial effects.

The outcomes of our study suggest that the influence of FAs on Aβ_1–42_ aggregation kinetics may not be strictly dependent on their degree of saturation. Both STA (18:0), a saturated FA, and AA (20:4), a polyunsaturated FA, were observed to delay Aβ aggregation. This finding challenges the assumption that FA effects on Aβ_1–42_ aggregation are based solely on saturation levels. STA is a common saturated FA in the human diet, known for its role in the structure and function of cell membranes.^41,42^ Despite concerns over saturated fats in cardiovascular health,^43,44^ STA might also play a role in the modulation of protein aggregation in neurodegenerative diseases.^45^ On the other hand, AA is integral to inflammatory responses^46,47^ and cell signaling pathways,^48,49^ with its derivatives participating in various physiological processes, including the modulation of synaptic function^50^ and neuroinflammation, which are critical in the context of AD. The similar impacts of STA and AA on Aβ_1–42_ aggregation suggest that the effects of FAs on amyloidogenesis are complex and may not be solely determined by their saturation status. The roles of AA and STA in the organism, coupled with their observed influence on Aβ_1–42_ kinetics, could inform a nuanced approach to therapeutic strategies, emphasizing the multifaceted roles of these FAs in cellular processes and disease progression. It could imply that other structural features or mechanisms may underlie the modulatory effects of other FAs. Unraveling these mechanisms could be key to developing new interventions for amyloid-related conditions such as AD, moving beyond saturation as the primary factor.

The unique capabilities of AFM-IR played a pivotal role in our study, enabling us to analyze individual Aβ_1–42_ aggregates and gain insights that would be challenging to obtain using standard methods like CD and FTIR. Unlike CD and FTIR, which measure the infrared spectra from all aggregates present in a sample, thus providing an averaged view, (Figure S1) AFM-IR offers the distinct advantage of studying single particles. This specificity allowed us to observe and record subtle variations in the secondary structure of individual Aβ_1–42_ oligomers or fibrils at different stages of formation, something that would be obscured in the data from CD or FTIR.

AFM and AFM-IR-based analysis of Aβ_1–42_ aggregates formed at different stages of formation provides a comprehensive view of how FA saturation influences amyloid β aggregation. AFM reveals highly consistent morphology of oligomers formed at the early stage of protein aggregation. Specifically, round-shaped oligomers were found in all samples, regardless of FA present at the stage of Aβ_1–42_ aggregation. This morphological uniformity suggests a fundamental and inherent characteristic in the early formation of Aβ oligomers. However, AFM-IR revealed subtle yet important differences in the secondary structure of these oligomers. Aβ_1–42_ aggregates formed in the absence of FAs predominantly had parallel β-sheets, a pattern that was slightly altered in FA-treated samples. Notably, while most FAs led to a decrease in parallel β-sheet content, the presence of DGLA and VA resulted in no change in the secondary structure. This deviation in structural composition among different FAs, irrespective of their saturation levels, implies a nuanced influence of FAs on amyloidogenesis.

We found elongated fibrils alongside the oligomers at the late stage of Aβ_1–42_ aggregation. AFM-IR revealed that these fibrils exhibited a higher amount of parallel β-sheets compared to the oligomers, indicating a structural evolution as the aggregation process advanced. This increase in parallel β-sheets suggests a maturation of the aggregate structure over time, possibly correlating with changes in the pathological potential of the aggregates. We also found slight variations in the parallel β-sheet content in the Aβ_1–42_ fibrils formed in the presence of different FAs. Thus, we can conclude that FAs can alter the secondary structure of Aβ aggregates. However, this effect is not overtly linked to their saturation level. That suggests that the interplay between FAs and Aβ is a complex process that cannot be solely explained by the saturation level of the FAs.

Using LDH, we observed a significant reduction in cytotoxicity in aggregates formed in at the late stages of Aβ_1–42_ aggregation the presence of certain FAs. Intriguingly, this decrease in cytotoxicity showed a correlation with the degree of unsaturation in the FAs. Specifically, Aβ_1–42_ fibrils formed in the presence of FAs with fewer unsaturated bonds demonstrated significantly lower cytotoxicity compared to Aβ_1–42_ fibrils grown in the presence of FAs with a greater number of double bounds. Thus, the chemical structure of FAs, particularly their saturation level, plays a critical role in modulating their protective effects against Aβ-induced cytotoxicity. This insight could be pivotal in developing dietary or pharmacological interventions targeting amyloid pathologies. It should be noted that Aβ_1–42_ oligomers formed at the early stages of protein aggregation in the presence and absence of FAs exerted similar toxicity. Recently reported results reported by Thomas and co-workers demonstrated that Aβ_1–42_ oligomers formed in the presence of AA (20:4) had far more deleterious effects on learning abilities and expression of AMPA receptors compared to oligomers formed in the lipid-free environment.^51^ These results suggest that oligomers formed by Aβ_1–42_ in the presence of FAs at the later stages of protein aggregation could exert comparably to Aβ_1–42_ fibrils higher cytotoxicity compared to the oligomers formed in the FA-free environment.

The JC-1 assay results, indicating the impact of FAs on mitochondrial membrane potential, further complement these findings. The presence of FAs did not significantly alter the mitochondrial membrane potential, suggesting a minimal impact on cellular health in terms of mitochondrial function, even in the presence of Aβ aggregates. Maintaining mitochondrial membrane potential is crucial, especially in the context of neurodegenerative diseases where mitochondrial dysfunction is a key pathological feature.^52^ The ability of FAs to preserve mitochondrial function amidst the challenge posed by amyloid aggregates points to their potential as neuroprotective agents. It should be noted that cell toxicity assays provide only partial understanding of the toxicity of amyloid aggregates. Subsequent *in vivo* studies have to be employed to fully understand the effect of FAs on amyloid development in AD patients.^51^

## Conclusions

In conclusion, our study reveals that the interaction between FAs and Aβ_1–42_ aggregation is a multifaceted process, transcending the simplistic notion of FA saturation levels as the primary modulatory factor. Both saturated and unsaturated FAs, such as stearic acid (STA) and arachidonic acid (AA), demonstrated a capacity to influence Aβ1–42 aggregation kinetics, challenging the conventional perspective that focuses solely on the degree of saturation. Our findings, supported by AFM and AFM-IR analyses, show that FAs can impact the morphological and structural characteristics of Aβ aggregates, irrespective of their saturation status. Moreover, the observed variations in cytotoxicity and the preservation of mitochondrial membrane potential in the presence of different FAs underscore the complexity of their role in amyloid pathologies. These insights suggest that factors beyond mere saturation levels of FAs are at play in modulating amyloidogenesis and its associated cytotoxicity. This research not only contributes to a deeper understanding of the relationship between dietary fats and neurodegenerative diseases but also opens new avenues for therapeutic approaches targeting amyloid-related conditions, emphasizing the nuanced roles of FAs in cellular processes and disease progression.

## Materials and Methods

### FA Stock Preparation

FAs were dissolved in DMSO to reach the final concentration of 40 mM and sonicated for 30 min. The solution was then diluted to 400 μM in 1× PBS and sonicated for an additional hour to ensure uniformity.

### Protein Aggregation

Synthetic human Aβ_1–42_ (AnaSpec, Cat.No AS-20276) was initially dissolved in 1 mL of HFIP (Across Organics, code 445820500) at a concentration of 1 mg/mL and allowed to incubate for 15 min. Subsequently, the HFIP was removed via evaporation under a stream of N_2_, resulting in the formation of a peptide film. This film was then reconstituted in 1× PBS at pH 7.4, with continuous vortexing while maintaining the mixture on ice, ultimately yielding a final concentration of 150 μM. The resulting samples were composed of 60 μM Aβ_1–42_ and 240 μM of FAs and were subjected to incubation at 25 °C under quiescent conditions for further analysis of protein aggregation.

### Kinetic Measurements

To assess the kinetics of protein aggregation, a Thioflavin T (ThT) fluorescence assay was employed. The samples were combined with ThT to achieve a final ThT concentration of 25 μM. The measurements were performed in a 96-well plate, maintaining quiescent conditions, at a temperature of 25 °C, using a Multimode microplate reader (Tecan Spark). Excitation was set at 450 nm, and emission signals were collected at 495 nm. Fluorescence readings were recorded at 5 min intervals throughout the experiment.

### AFM and AFM-IR

For AFM and AFM-IR imaging, 5 μL of solutions containing protein aggregates were deposited onto silicon wafers and allowed to sit for 3 min. Subsequently, the silicon wafers underwent a gentle rinse with DI water and were then dried using a stream of N_2_. Imaging was conducted utilizing a Nano-IR3 system (Bruker, Santa Barbara, CA) equipped with a QCL laser. Contact-mode AFM tips (ContGB-G AFM probe, NanoAndMore) were employed for capturing images, spectra, and IR maps. Spectra were obtained from individual oligomers or fibrils, and each spectrum represented an average of three spectra. Approximately 20–30 single aggregates were analyzed for each sample. The raw spectra underwent processing through a 10-point smoothing filter in Analysis Studio v3.15 and were subsequently normalized based on average area. Spectral fitting was executed using GRAMS/AI 7.0 (Thermo Galactic, Salem, NH). The spectra obtained from each sample were then averaged, and subpeaks corresponding to protein secondary structures were estimated via peak deconvolution. Within the amide I region (1600–1700 cm^–1^), peaks related to parallel β-sheet (1610–1640 cm^–1^), unordered protein (1641–1685 cm^–1^), and antiparallel β-sheet (1675–1697 cm^–1^) were fitted. The percentage of each secondary structure was determined by calculating the area under the curve. The peak area for each secondary structure was normalized to the total peak area within the amide I region, and the corresponding percentages of the amide I band region for each secondary structure were reported in this study.

### Circular Dichroism (CD)

Circular Dichroism (CD) measurements were conducted using a J-1000 CD spectrometer (Jasco, Easton, MD) operating at 25 °C. For each sample, triplicate measurements were taken across the wavelength range of 195–250 nm.

### Attenuated Total Reflectance Fourier-Transform Infrared (ATR-FTIR) Spectroscopy

Two μL aliquots of the sample were applied onto an ATR crystal and permitted to air-dry at room temperature. Subsequently, FTIR spectra were recorded utilizing a Spectrum 100 FTIR spectrometer (PerkinElmer, Waltham, MA).

### Cell Toxicity Assays

The N27 rat dopaminergic neuron cell line was cultured to RPMI 1640 Medium (Thermo Fisher Scientific, Waltham, MA) supplemented with 10% fetal bovine serum (FBS) (Invitrogen, Waltham, MA) in 96-well plates (10,000 cells per well) at 37 °C under 5% CO_2_. After 24 h of incubation, the cells were found to be fully adherent. For the lactate dehydrogenase (LDH) assay, 100 μL of the medium was replaced with 100 μL of RPMI 1640 Medium containing 5% FBS and 10 μL of the protein samples with final concentration of protein and FA of 6 and 24 μM respectively. The concentration of FBS was reduced to lower the baseline absorbance level of analyzed samples. After 24 h of incubation, the amount of LDH released into the cell culture medium was quantified using the nonradioactive CytoTox 96 cytotoxicity assay kit (G1781, Promega, Madison, WI). LDH is a cytosolic enzyme that is released into the surrounding cell culture medium upon damage to the plasma membrane. The concentration of LDH was determined by measuring the conversion of lactate to pyruvate via NAD^+^ reduction to NADH, which is utilized to reduce a tetrazolium salt into a red formazan product that has an absorption maximum at 490 nm. The level of formazan directly correlated with the amount of LDH released, which in turn represented the toxicity of the protein aggregates toward N27 cells.

Following a 3 and 48-h incubation period, a JC-1 assay was performed. Cells were cultured in 48-well plates (30,000 cells per well) at 37 °C under 5% CO_2_. After 24 h of incubation, the cells were found fully adherent, the cell culture medium was replaced with fresh RPMI 1640 with 10% FBS. The sample was added to obtain the same concentration as for the LDH assay. After 24 h of incubation with the sample, JC-1 reagent (M34152A, Invitrogen) was added to the cells to obtain 50 μM of final concentration and incubated at 37 °C in a 5% CO_2_ environment for 30 min. Following the removal of the supernatant, after treatment with trypsin, the cells were resuspended in 200 μL of the 1× PBS, pH 7.4. Sample measurements were obtained using the green channel (*k* = 488 nm) of an Accuri C6 Flow Cytometer (BD, San Jose, CA). The percentage of cells exhibiting JC-1 staining was determined relative to positive control with carbonyl cyanide *m*-chlorophenyl hydrazone.

### Fluorescence Microscopy

N27 rat dopaminergic neuron cells were cultured in 35 mm dishes with an optical bottom (Cellvis, Cat. No. D35–10–1.5-N) at a density of 300,000 cells per dish. The cell culture was maintained in RPMI 1640 Medium (Thermo Fisher Scientific) supplemented with 10% fetal bovine serum (FBS) (Invitrogen, Waltham, MA) at 37 °C in a 5% CO_2_ environment. After a 24-h incubation, during which the cells adhered to the well surface, the cell culture medium was replaced with fresh RPMI 1640 Medium and 10% FBS, which also included the protein samples. Subsequently, JC-1 reagents were added to achieve final concentrations of 5 and 50 μM, respectively. The cells were then incubated for 20 min at 37 °C in a 5% CO_2_ environment. To complete the staining process, 1 drop of NucBlue Live Cell ReadyProbes (Invitrogen, Cat. No. R37605) was added to each sample and incubated for an additional 5 min at 37 °C in 5% CO_2_. Fluorescence images were captured using the EVOS M5000 Imaging System (Invitrogen), equipped with an Olympus UPIanApo 100*x*/1.35 oil iris ∞/0.17 objective and filters for blue, red, and green channels.

## Supporting Information Available

Kinetic Modeling Equations for Aβ1–42 Aggregation in the Presence of FAs (Table S1); CD and FTIR spectra of protein aggregates (Figure S1); histograms of cytotoxicity of FAs (Figure S2), and ThT kinetics of protein aggregation (Figure S3) (PDF)
