# Supplementary material for: Nanoscale Structural Characterization of Amyloid β 1–42 Oligomers and Fibrils Grown in the Presence of Fatty Acids
Source: ACS Chem Neurosci. 2024 Sep 2;15(18):3344–53. doi: 10.1021/acschemneuro.4c00275 (PMC11413849; doi:10.1021/acschemneuro.4c00275)
Supplement: Supplementary file 1 — cn4c00275_si_001.pdf [file cn4c00275_si_001.pdf]

Nanoscale Structural Characterization of Amyloid Beta 1-42 Oligomers and Fibrils Grown in the Presence of Fatty Acids

Kiryl Zhaliazka[1] and Dmitry Kurouski*[1,2]

1. Department of Biochemistry and Biophysics, Texas A&M University, College Station, Texas 77843, United States

2. Department of Biomedical Engineering, Texas A&M University, College Station, Texas, 77843, United States

Correspondence: dkurouski@tamu.edu

Supporting Information

SI

	Equation
$A\beta_{1-42}$	-1.0452*exp(-0.20393*x)+1.008
	-0.97152*exp(-0.17054*x)+0.98865
	-0.9505*exp(-0.18082*x)+1.004
	-1.0262*exp(-0.13877*x)+1.0158
	-1.0378*exp(-0.20478*x)+1.009
	-1.0502*exp(-0.1951*x)+1.0096
$A\beta_{1-42}$:EPA (20:5)	-0.97591*exp(-0.17453*x)+1.0054
	-0.8475*exp(-0.11238*x)+0.99806
	-0.89454*exp(-0.14895*x)+0.99861
	-0.88477*exp(-0.17352*x)+0.98913
	-0.92789*exp(-0.18449*x)+1.0017
	-0.91314*exp(-0.15258*x)+1.0035
$A\beta_{1-42}$:DHA (20:6)	-0.95904*exp(-0.15945*x)+1.0034
	-0.91047*exp(-0.14825*x)+0.99194
	-0.85246*exp(-0.1586*x)+0.96285
	-0.80656*exp(-0.19037*x)+0.93757
	-1.0321*exp(-0.19685*x)+0.99795
	-0.81254*exp(-0.26831*x)+0.94647
$A\beta_{1-42}$:DGLA (20:6)	-0.86843*exp(-0.20374*x)+0.95216
	-0.89934*exp(-0.21048*x)+0.96171
	-0.88396*exp(-0.21903*x)+0.95628
	-0.868*exp(-0.18699*x)+0.98372
	-0.8501*exp(-0.17101*x)+0.97569
	-0.88652*exp(-0.18189*x)+0.98254

	Equation
$A\beta_{1-42}$:AA (20:4)	-0.88631*exp(-0.11248*x)+0.99351
	-0.9426*exp(-0.11349*x)+1.0055
	-0.88225*exp(-0.068353*x)+1.0012
	-0.99547*exp(-0.092264*x)+1.0308
	-1.0411*exp(-0.096517*x)+1.0377
	-0.92207*exp(-0.083225*x)+1.0044
$A\beta_{1-42}$:VA (18:1)	-0.87*exp(-0.19182*x)+1.0061
	-0.90121*exp(-0.12836*x)+0.98691
	-0.8966*exp(-0.16424*x)+0.95567
	-0.87346*exp(-0.18471*x)+0.98394
	-0.86632*exp(-0.12639*x)+1.0044
	-0.94274*exp(-0.26995*x)+0.93366
$A\beta_{1-42}$:EA (18:1)	-0.8411*exp(-0.078356*x)+0.992
	-1.0261*exp(-0.19354*x)+1.0088
	-1.0204*exp(-0.1916*x)+1.0089
	-0.81407*exp(-0.081482*x)+0.97968
	-1.0684*exp(-0.36441*x)+1.0025
	-0.86506*exp(-0.10609*x)+0.98871
$A\beta_{1-42}$:STA (18:0)	-0.88006*exp(-0.1239*x)+0.95683
	-0.76309*exp(-0.082198*x)+0.98873
	-0.9814*exp(-0.13367*x)+0.99002
	-0.83135*exp(-0.10899*x)+0.95528
	-0.99666*exp(-0.094482*x)+1.0149
	-0.74482*exp(-0.10063*x)+0.93462

Table S1: Kinetic Modeling Equations for Aβ1-42 Aggregation in the Presence of FAs

This table presents the set of equations used for fitting the aggregation kinetics of Aβ1-42 alone and in the presence of various FAs using MATLAB. The equations are derived to best represent the time-course data obtained from the Thioflavin T fluorescence assays, reflecting the rate and extent of amyloid fibril formation over time. Each equation is of the form $y = a * \exp(b * x) + c$, where y is the ThT fluorescence intensity, x is time, and a, b, and c are fitting parameters that describe the magnitude, rate, and baseline of the aggregation process, respectively.

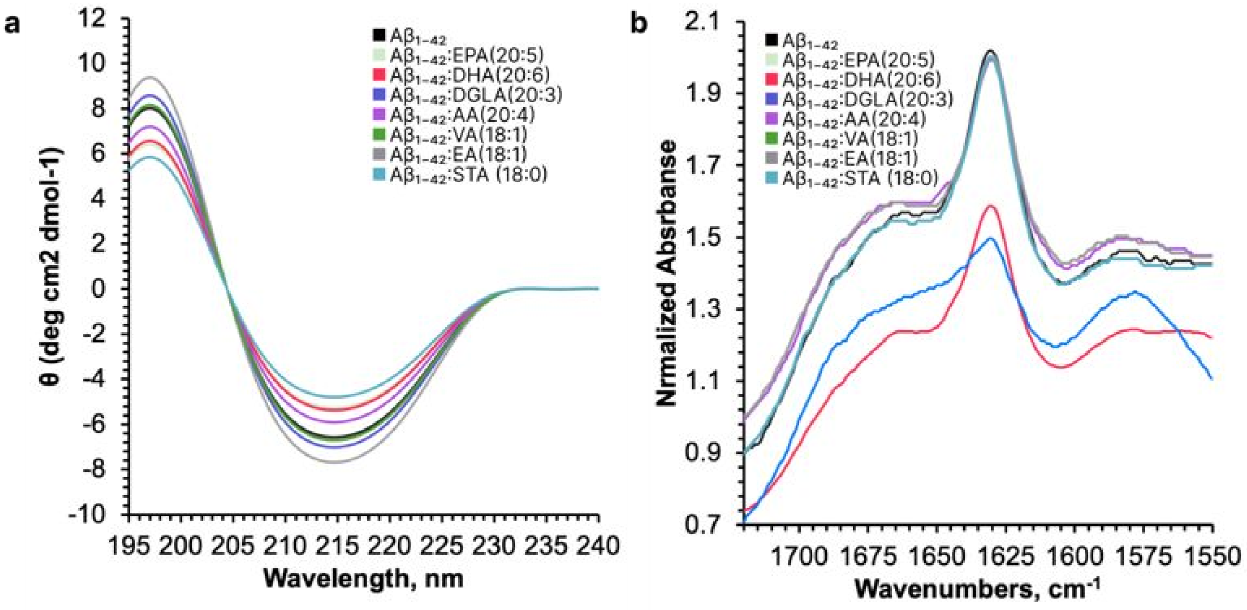

Figure S1: Spectroscopic Analysis of A$\beta_{1\text{-}42}$ Fibrils Grown in the Presence of Fatty Acids

(a) Circular dichroism (CD) spectroscopy profiles of A$\beta_{1\text{-}42}$ alone and in the presence of various FAs at 48 h of incubation, illustrating the molar ellipticity (θ) across the ultraviolet (UV) wavelength spectrum. This analysis captures the secondary structural elements of the peptides, with the characteristic minimum at approximately 218 nm indicative of β-sheet content. (b)Fourier-transform infrared (FTIR) spectroscopy absorbance spectra of A$\beta_{1\text{-}42}$ samples, normalized for comparison. The spectra display the amide I band region, which is sensitive to secondary structures, with a peak around 1625 cm^{-1} corresponding to the β-sheet structures.

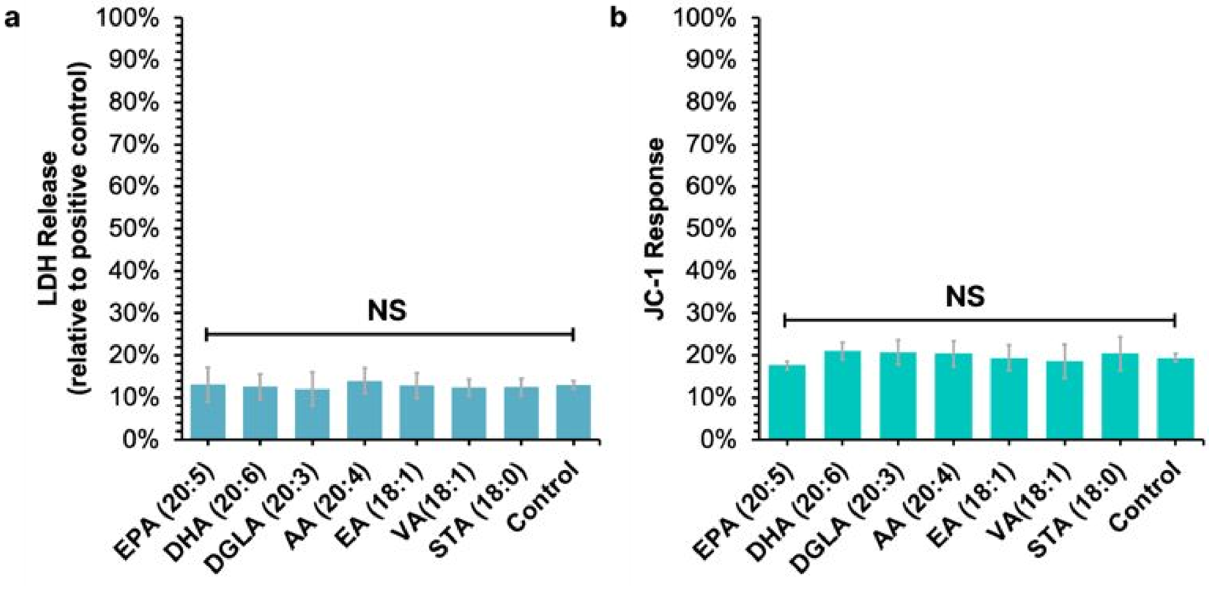

Figure S2: Evaluation of Fatty Acid Impact on Neuronal Cytotoxicity and Mitochondrial Function

(a) Bar graph displaying the relative lactate dehydrogenase (LDH) release from neuronal cells treated with various FAs in comparison to the control group. Cytotoxicity is expressed as a percentage relative to a positive control. The data were

analyzed using one-way ANOVA ($p>0.05$), indicating no significant (NS) differences between the treated groups and control.

(b) Bar graph showing the JC-1 assay response, assessing the mitochondrial membrane potential in the same treatment groups. The results were analyzed using one-way ANOVA ($p>0.05$), which revealed no significant (NS) differences, suggesting that mitochondrial function was not adversely affected by the FAs under the study conditions.

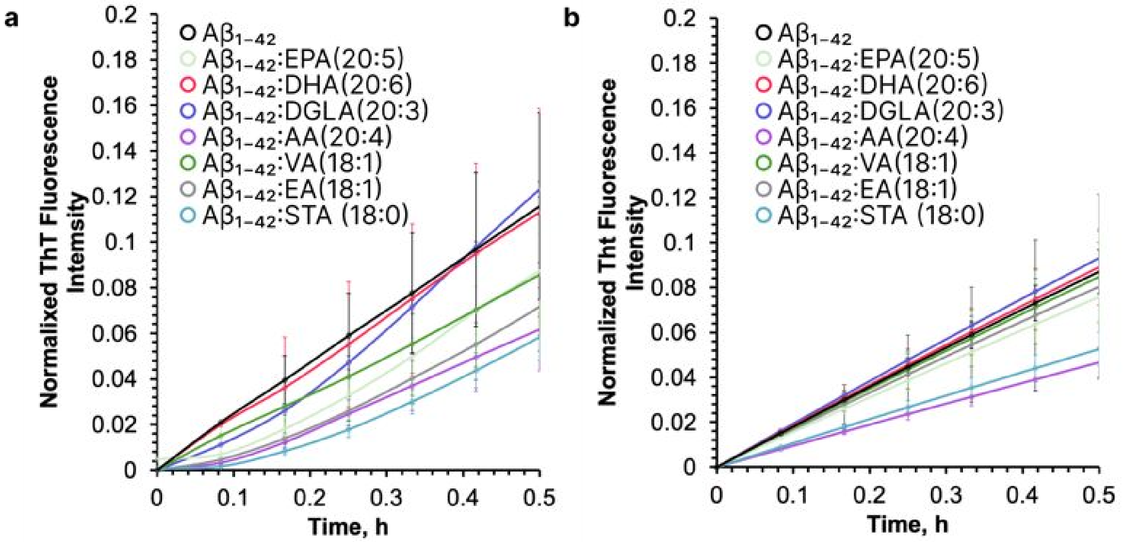

Figure S3: Influence of FAs on the Early Stage of ThT Aggregation Kinetics of A$\beta_{1\text{-}42}$. (a) The aggregation kinetics of A$\beta_{1\text{-}42}$ in the presence of different FAs, as indicated by normalized Thioflavin T fluorescence. (b) The same data were fitted using equations from Table S1.
